# Tackling overweight and obesity: does the public health message match the science?

**DOI:** 10.1186/1741-7015-11-41

**Published:** 2013-02-18

**Authors:** Katherine Hafekost, David Lawrence, Francis Mitrou, Therese A O'Sullivan, Stephen R Zubrick

**Affiliations:** 1 Telethon Institute for Child Health Research, Centre for Child Health Research, The University of Western Australia, PO Box 855 West Perth WA6872 Australia; 2 School of Exercise and Health Science, Edith Cowan University, Joondalup, WA, 6027, Australia

**Keywords:** Energy balance, obesity, public health, weight-loss intervention

## Abstract

**Background:**

Despite the increasing understanding of the mechanisms relating to weight loss and maintenance, there are currently no validated public health interventions that are able to achieve sustained long-term weight loss or to stem the increasing prevalence of obesity in the population. We aimed to examine the models of energy balance underpinning current research about weight-loss intervention from the field of public health, and to determine whether they are consistent with the model provided by basic science. EMBASE was searched for papers published in 2011 on weight-loss interventions. We extracted details of the population, nature of the intervention, and key findings for 27 articles.

**Discussion:**

Most public health interventions identified were based on a simple model of energy balance, and thus attempted to reduce caloric consumption and/or increase physical activity in order to create a negative energy balance. There appeared to be little consideration of homeostatic feedback mechanisms and their effect on weight-loss success. It seems that there has been a lack of translation between recent advances in understanding of the basic science behind weight loss, and the concepts underpinning the increasingly urgent efforts to reduce excess weight in the population.

**Summary:**

Public health weight-loss interventions seem to be based on an outdated understanding of the science. Their continued failure to achieve any meaningful, long-term results reflects the need to develop intervention science that is integrated with knowledge from basic science. Instead of asking why people persist in eating too much and exercising too little, the key questions of obesity research should address those factors (environmental, behavioral or otherwise) that lead to dysregulation of the homeostatic mechanism of energy regulation. There is a need for a multidisciplinary approach in the design of future weight-loss interventions in order to improve long-term weight-loss success.

## Background

Overweight, obesity and their associated chronic diseases are significant global public health issues with considerable costs to both the individual and the community. Much research has been devoted to identifying the primary causes, methods of prevention, and effective treatments for these conditions. Numerous research projects, public health campaigns, and population interventions have sought to quantify the energy gap that has led to this epidemic, to identify its primary environmental, behavioral, and genetic causes, and to determine effective interventions that could be applied to the general population. Despite these efforts, effective long-term interventions remain elusive, and treatments have been labeled palliative [1].

Public health research, recommendations, and interventions relating to the treatment and prevention of overweight and obesity are often based on a simple model of energy balance. This model suggests that change in body weight is equal to energy intake minus energy expenditure, and is often used to show how reductions in caloric intake and/or increases in physical activity will result in successful weight loss that can be maintained over the long term. A common assumption is that energy intake and energy expenditure can be independently modified, through changes in caloric consumption and physical activity, to effect changes in energy balance. For instance, a person could achieve a negative energy balance and thus weight loss, by either reducing their caloric intake or increasing their level of physical activity, or both. This is based on the faulty assumption that each component of energy intake and expenditure can be modified without compensatory changes in other components. However, as is known from much research in the fields of biochemistry and physiology, energy input and expenditure are interdependent and regulated at several levels. This body of research details a far more intricate and complex model of how multiple feedback mechanisms operate to homeostatically regulate energy balance and thus maintain body weight within a relatively narrow range [2]. For instance, when caloric intake is cut the body naturally responds by both stimulating hunger and reducing basal energy expenditure so that less energy is expended. Similarly, a period of increased physical activity can be followed by a period of both increased hunger and increased fatigue, resulting in reduced energy expenditure at other times during the day.

However, despite this knowledge, public health interventions and dietary guidelines largely neglect this evidence, instead persisting with interventions and recommendations based on an unmodified view of the energy-balance model. As noted by Sorensen [3], ignoring dependencies that regulate energy intake and expenditure leads to a misunderstanding of the causes of weight gain and thus design of inappropriate interventions that are often unsuccessful in the long term. Using the simple energy-balance model leads researchers to ask the basic question, why do people eat too much, exercise too little and thus gain weight? However, a more nuanced understanding of energy regulation would lead to posing the question, what personal, genetic, environmental, and other factors cause dysregulation of energy balance?

To examine the consistency between the basic energy-balance model used in public health and the more complex homeostatic feedback model developed from human biochemistry and physiology, we reviewed recently published public health research that investigated methods to achieve weight loss or prevent weight gain. The conceptual or theoretical basis underpinning each study was assessed to examine whether the strategies used were based on the basic energy-balance model or incorporated feedback mechanisms.

## Current public health intervention research

To identify relevant research we searched EMBASE for public health interventions and trials in which researchers manipulated energy intake and/or energy expenditure with the aim of achieving weight loss or preventing weight gain. The key search terms were 'calorie restriction', 'energy', 'weight loss intervention', 'energy restriction', 'physical activity', 'overweight', 'obesity', 'diet', 'exercise' and 'public health'. In addition, key journals in the fields of public health research, nutrition, and obesity were hand-searched for relevant articles.

Although our search was comprehensive, it was not exhaustive. In order to focus the review, studies were included only when the primary outcome was a measured change in weight. Additionally, non-English language studies, pharmaceutical or surgical trials, gene studies, or studies involving animal models, weight-loss supplements, pregnant women, or populations defined by existing disease were excluded, as were review articles and research proposals. The search was limited to studies published in 2011, in order to limit the size of the review and to focus on current approaches.

Article titles and abstracts were assessed, and full text versions were obtained for the articles meeting these criteria. For each study we extracted details of the nature of the intervention and attempted to summarize, to the extent described, the underlying biological model. We gave particular focus to whether the model underpinning the intervention included homeostatic feedback mechanisms, and how they were incorporated into the intervention design.

In total, 27 interventions assessing potential methods for weight loss or for weight-gain prevention were identified. These studies aimed to evaluate interventions that could be used to address levels of overweight and obesity in general population groups of adults and/or children (for key study characteristics, see Additional file 1).

## Discussion

Many of the identified articles acknowledged the complexity of the development of overweight and obesity, and highlighted the need for a multifaceted treatment approach. Treatment approaches investigated in these studies involved reducing energy intake and increasing physical activity in order to achieve a negative energy balance. Additionally, behavioral counseling, support, or education components were included to increase program adherence and maximize participant success.

Reductions in caloric intake were achieved through a variety of methods. Commonly identified methods included the provision of specific caloric goals [4-10], and use of pre-packaged meals or meal replacements to limit caloric consumption and restrict energy intake [4,7,11,12]. Of the 27 studies, 17 provided nutrition-education components, with common themes being reducing fat intake, limiting high-energy snack foods, reducing or eliminating consumption of sweetened beverages [6,12-18], and controlling portion sizes [12,13].

In addition to targeting diet, interventions aimed to increase energy expenditure in order to reach a negative energy balance. With the exception of two studies, where insufficient detail was provided about the physical activity [19,20], all studies aimed to increase the physical-activity levels of participants. Interventions used a variety of methods to achieve this aim. Common methods included providing specific exercise goals [4,9,15,21-24], requiring participants to complete structured exercise programs [6,7,11,13,21,25], or encouraging participants to increase their daily levels of physical activity without specific exercise goals. In addition, novel methods for increasing physical activity, such as locking devices for televisions [12] and use of active video games [26], were investigated. Tools for activity tracking such as online reporting of activity or pedometers were used to establish whether activity goals were achieved and to monitor energy expenditure [8,9,14,15,17,27]. Reducing sedentary behaviors was a common theme. In particular, reducing screen time in an effort to increase total energy expenditure was often encouraged [15,25,26,28].

A common element of the identified studies was the inclusion of motivational and/or educational components with the aim of maximizing program adherence. Examples included online, telephone or in-person counseling sessions; involvement of parents or family members; behavioral-modification support; and use of monetary and other incentives to encourage program adherence. As mentioned previously, education regarding healthy dietary and exercise behaviors was frequently provided [15,16,18,22-25,27-31], and some interventions specifically included information about the need to balance energy intake and energy expenditure to achieve weight loss [8,27].

Even though the identified studies included a range of age groups, study periods, and methods, they appeared to share the same underlying causal basis: that achieving a negative energy balance, through increases in exercise energy expenditure and reductions in energy intake, would result in successful weight loss. Although the studies typically suggested that the causes of excess weight gain are complex, and stated that treatment approaches need to be multifaceted, they nonetheless appeared to adhere to an unadjusted model of energy balance. Interventions typically prescribed reductions in total caloric intake, increases in physical activity, and reductions in sedentary behaviors in order to achieve a negative energy balance and induce weight loss.

Almost all the identified interventions focused on reducing energy intake, increasing physical activity, and reducing sedentary behaviors underpinned by an energy-balance model assuming independence between energy intake and expenditure, with little consideration of homeostatic feedback mechanisms. Although these interventions typically promoted healthy behaviors, the primary aim of all identified studies was weight loss or prevention of excess weight gain. Although statistically significant short-term weight and/or fat losses were achieved in many of the studies (see Additional file 1), weight change was often small and weight regain was evident in a number of studies [6,15,17,23]. This finding suggests that, although restriction of energy intake and increased physical activity energy expenditure can achieve short-term weight loss, it does not provide a successful long-term treatment for excess weight as many people regain the lost weight as the body adapts to new levels of energy intake and expenditure. This is supported by the existing literature, which has shown that early weight loss, achieved by energy restriction and increased activity, is rarely maintained over the long term [32,33]. Although any net weight loss achieved by interventions could be considered positive, it is currently unclear whether short-term loss and subsequent weight gain is beneficial or detrimental to long-term health. Maximizing an individual's ability to lose and maintain weight should be the aim of any weight-loss intervention.

### Understanding from basic science

Research from the fields of biochemistry and human physiology, which provides a more detailed model of energy balance, offers some insight into why many weight-loss interventions have little long-term success and poor program adherence. Although not offering a complete understanding of the biological mechanisms at play, this body of research has identified a number of mechanisms that act in both the short and long term in response to fluctuations in energy balance. These mechanisms allow energy intake to be matched to expenditure, and permit the maintenance of body weight within a relatively narrow range. Although in the short term, food intake and energy expenditure are often influenced by situational factors, over longer time periods numerous neural and hormonal mechanisms operate to regulate body weight. As stated by Sumithran and colleagues, 'body weight is centrally regulated with peripheral hormonal signals released from the gastrointestinal tract, pancreas and adipose tissue integrated primarily in the hypothalamus to regulate food intake and energy expenditure' [34]. These integrated controls seem to favor protection against weight loss rather than prevention of fat accumulation. Although compensatory responses such as increases in resting energy expenditure and decreased appetite in response to weight gain have been identified [35,36], the high levels of overweight and obesity within western populations suggest that these homeostatic feedback signals are relatively easily over-ridden in terms of weight gain. However, in the context of weight loss, these homeostatic mechanisms have significant implications. Their operation means that although negative energy balance is likely to result in initial weight loss, as evident in many of the trials and interventions outlined above, this weight-loss success is likely to be limited over the long term. Restrictions in caloric intake and/or increases in physical activity are likely to be matched by behavioral, metabolic, neuroendocrine, and autonomic changes that will limit long-term success [37,38]. These changes, which may result in increased hunger and fatigue, are likely to make adherence to strict caloric and exercise goals challenging for participants, and therefore necessitate intensive education and counseling in order to maintain participant numbers in weight-loss interventions.

In addition to outlining the mechanisms affecting weight change, the quantitative aspects of changes in body weight and energy balance have been described. Hall and colleagues demonstrated that, if energy intake is increased by a fixed amount to create an initial positive energy imbalance, the storage of excess energy in either additional lean or fat mass requires additional energy to maintain that additional tissue. As a result, energy expenditure rises to match the increased energy intake and a new equilibrium level is achieved. However, Hall and colleagues state that even if energy intake and physical activity are both independently set, the change in body weight is less than that predicted by this model, owing to the effect of feedback mechanisms that adjust basal energy expenditure to balance energy availability [39,40].

### Factors affecting homeostatic regulation of energy

A number of environmental factors that are potentially problematic for control of body weight, and therefore have implications for intervention design, have also been identified. For example, the hedonistic qualities or hyperpalatability of high-sugar or high-fat products have been suggested to encourage reward driven eating and may over-ride biological controls [41]. The unique metabolic and hormonal effects of chronic and high consumption of carbohydrates, particularly refined carbohydrates, fructose, and sugar-sweetened beverages, have been linked with low satiation, poor appetite control, and a lack of compensation for calories consumed over the short and long term. These factors, which have an increasing role in diet over recent decades, may contribute to excess weight gain and interrupt weight loss and weight maintenance. The role of dietary quality in body-weight control is emphasized by Mozaffarian and colleagues who state that, 'specific dietary and lifestyle factors are independently associated with long-term weight gain...' and 'Individual and population-based strategies to help people consume fewer calories may be most effective when particular foods or beverages are targeted for decreased (or increased) consumption' [42]. In addition, Bray illustrated differences in weight loss and gain with differences in the macronutrient composition of the diet [43].

A range of studies and popular media support the use of specific dietary patterns for weight loss and maintenance. No interventions based on these principles met the criteria for inclusion in our review. There are various schools of thought on how diets may be modified in ways that effect the regulation of energy balance. These include carbohydrate-restricted diets, which have been linked with favorable metabolic outcomes for weight loss, weight maintenance, and health [44-46]. Carbohydrate restriction may achieve, for example, improved glycemic and insulin control, increased mobilization and utilization of lipid substrates, inhibition of lipogenesis, favorable changes in circulating fatty acids, and improvements in atherogenic dyslipidemia, lipoprotein markers and inflammation [47-49]. Low-glycemic-index and low-sugar diets have been proposed in an attempt to regulate postprandial glycemia and hyperinsulinemia [50] and low-fructose diets have been suggested with the aim of eliminating the potential negative effects of fructose consumption on energy regulation [51]. A number of studies have explored differences between these dietary patterns and conventional low-fat diets [52-55]. However, in spite of this body of evidence carbohydrate-restricted diets continue to be on the fringes of public health intervention research and remain noticeably absent from dietary guidelines [48,56]. Although many of the studies considered in this review encouraged reductions in high-sugar products and in particular sugar-sweetened beverages, this appeared to be related to reducing caloric intake rather than to their effect on the regulation of metabolism. The inclusion of any dietary protocols based on a complex model of energy balance would be a significant step forward in public health intervention research. Adoption of these approaches should lead to better understanding of which types of diets work best, and for whom.

### Implications for population health interventions

The failure of research in the field of public health to incorporate the concept of homeostatic feedback mechanisms into interventions is reflected in the current dietary guidelines, public health policy, and population-wide interventions aimed at targeting overweight and obesity. For example, the current dietary guidelines in the USA state 'To curb the obesity epidemic and improve their health, many Americans must decrease the calories they consume and increase the calories they expend through physical activity'[57]. Australian recommendations mirror this stance, suggesting that individuals should lower the energy density of their diet through restrictions in fat intake and increased intake of fruit and vegetables, increase their physical activity, and reduce sedentary behavior in order to prevent or address excess weight gain [58]. Public health interventions such as *Let's move *in the USA, *Healthy Weight Healthy Lives *and *Change For Life *in the UK, and] *Eat Smart Play Smart, Time2bHealthy, Be Active Eat Well*, and *Move And Munch *interventions in Australia focus on changing energy balance. Again, it is often suggested that this reduction in energy intake is achieved by decreasing intake of energy-dense foods and increasing active behaviors in order to combat excess weight gain in the population. Although these changes may promote good health, the existing evidence suggests that merely reducing energy intake and increasing energy expenditure, without acknowledging the homeostatic response to a negative energy balance, is unlikely to successfully reduce levels of overweight and obesity in the population. Additionally, it is possible that losing and regaining excess weight may have negative psychological and physical health consequences which should be considered in the implementation of future weight-loss interventions [59].

A flow-on effect of using the simple energy-balance model to underpin intervention design was seen in the large number of interventions that incorporated psychological or behavioral counseling components. A common response to the repeatedly observed failure of interventions based on the simple energy-balance model to achieve long-term weight loss, or prevent weight gain, is to assume that people are not following the prescriptions of the intervention. Thus, interventions need to incorporate more strategies to promote behavior modification. We found little evidence that the interventions we reviewed were designed in collaboration with expertise in physiology or metabolism. Dietary and physical activity guidelines are often taken as the only basic-science input needed to design interventions. In most cases these guidelines historically were developed to address risks for other chronic conditions, such as cardiovascular disease, and do not reflect the current understanding of energy balance [56]. An important first step in future intervention research should be for public health researchers and advocates to form collaborations with those who have expertise in physiology and metabolism, in order to develop more appropriate protocols. Interventions should assess the value of dietary patterns and exercise protocols that have been identified to assist in weight loss and maintenance, rather than continuing to test existing guidelines and recommendations.

### Directions for future research

Public health interventions that promote dietary improvements, such as limiting the intake of sugar-sweetened beverages and increasing the levels of physical activity, may have a range of health benefits. For instance, increased physical activity and fitness reduces the risk of cardiovascular disease. Nevertheless, explicitly promoting reduced energy consumption and increased expenditure as the appropriate means by which to achieve weight loss poses ethical challenges. Despite the extensive literature on their long-term ineffectiveness, interventions based on this simplistic understanding of energy balance continue to be advocated under the assumption that previous interventions have not been pursued sufficiently vigorously or that participants have failed to follow the prescriptions of the intervention. It is very possible that some people who follow these interventions but fail to lose and maintain weight may become discouraged and discontinue the intervention, thus missing out on other possible health benefits. Continuing to promote a model that is unlikely to be successful in the longer term, and may result in individuals becoming discouraged, is both unproductive and wasteful of resources that could be better spent on investigating more plausible alternatives to improving weight control.

Although simple models are useful in describing or explaining complex systems, particularly for use in the general population where specialist knowledge and understanding is limited, if the model fails to accurately describe how the system operates then it is both misleading and unhelpful. Weight-loss interventions that can be implemented at a population level should be based on biologically plausible models of energy balance. Such a model could not only improve the success of interventions but could provide a basis for research into potentially modifiable factors that influence the homeostatic control of body weight. For instance, from the diet perspective this model might suggest considering, in addition to total kilojoules, the effect of diet composition, including nutrient profile and interaction between nutrients and hormones regulating energy balance. Regarding physical activity, the mode, type, duration, and intensity of exercise on the homeostatic control mechanisms could be considered. Jointly, these factors may facilitate understanding of key causes and treatments of excess weight gain. In order to develop such a model, a multidisciplinary approach that incorporates knowledge and understanding from the fields of public health and the basic sciences should be adopted.

In many of the interventions we reviewed, the basis for the dietary intervention appeared to be the dietary guidelines rather than active collaboration with experts in the appropriate basic sciences. The reliance on dietary guidelines to inform the scientific basis of interventions may be a significant factor in holding back progress as there are very few public health interventions testing alterative models. Although dietary guidelines may be a useful tool for informing the public on the most appropriate diets for overall health and wellbeing, these guidelines should not be used as the sole basis for ongoing research in the field of obesity treatment and prevention. A range of alternative interventions, including carbohydrate restricted, low glycemic index, and low fructose, have been proposed based on more plausible models of energy balance. Although there is existing research, as described above, indicating potential benefits of one or more of these carbohydrate restricting approaches, this research has not yet been translated into comprehensive intervention trials. More research is needed to determine how public health interventions can be developed from these models. The focus of future public health research should be on the development of large-scale, long-term prospective studies that test dietary and exercise protocols that have been shown to be beneficial to weight loss and maintenance.

Multidisciplinary collaborations could be encouraged by a number of means. For example, ensuring that funding or grant panels considering proposals for future intervention research include members trained in human biochemistry and physiology as well as public health advocates. This may assist in the development and implementation of appropriate research designs and methods that are underpinned by a complex model of energy balance.

Although we were unable to identify any public health interventions using more biologically plausible models of energy regulation, more recently there is evidence to suggest a shift towards more complex models of energy balance underpinning interventions in the field of public health. For example, a recent series published in the *New England Journal of Medicine *examined the role of sugar-sweetened beverages on weight change [60,61]. It seems the authors investigated these interventions because of their belief that sugars consumed in liquid form may bypass the homeostatic regulation of satiety and reduce the insulin response. The biological model underpinning these interventions remains underdeveloped, addressing only a small component of dietary carbohydrate restriction. However, it is a positive sign to see some consideration of models that are not based on restriction of caloric intake.

In general, dietary carbohydrate restriction offers an alternative to the energy-balance principle and has shown good results in comparison to low-fat diets particularly in people with metabolic syndrome. The effects are attributed not only to spontaneous reduction in consumption but also to energy inefficiency, popularly known as 'metabolic advantage'. Researchers in this field have demonstrated the fallacies in the thermodynamic analysis that is supposed to provide support for the energy-balance model [45,62]. Perhaps because this concept explicitly challenges the 'calories in, calories out' model, we were unable to find any public health interventions based on carbohydrate restriction meeting our search criteria and published in 2011.

## Summary

The results of this review show the discord between the significant body of research describing the metabolic and physiologic underpinnings of weight gain and loss, and the most recent population-based interventions designed to address the obesity epidemic. We found little evidence of multidisciplinary collaborations and much evidence of a lack of translation between advances in understanding of the basic science and the basis of these interventions.

As noted by Bray, 'obesity is a chronic, relapsing, neurochemical disease' [1]. This concept, based on a biological understanding of homeostatic mechanisms, should have led to a change in the framing of key questions of obesity research to address those factors (environmental, behavioral or otherwise) that lead to dysregulation of the homeostatic mechanism of energy regulation. Currently, most research seems to address the question of what factors lead people to eat too much and exercise too little. There is an evident need for a multidisciplinary approach in the design of future weight-loss interventions in order to frame the research questions appropriately. Developing and testing interventions that are based on biologically plausible mechanisms is an important step forward in developing effective interventions to combat obesity and its associated metabolic diseases.

## Competing interests

The authors declare that there are no competing interests.

## Authors' contributions

KH, DL, and FM conceived the design of the study. KH undertook the literature search and reviewed the papers. KH drafted the manuscript. All authors contributed to the writing and approved the final manuscript.

## Author information

KH is an analyst at the Telethon Institute for Child Health Research (TICHR). DL is a research professor at The University of Western Australia (UWA) and a senior statistician at TICHR. FM is a senior analyst at TICHR. TOS is an accredited practicing dietician and senior researcher affiliated with TICHR, and a senior lecturer in nutrition and dietetics at Edith Cowan University. SZ is a Winthrop Professor in the Centre for Child Health Research at UWA, and head of the Division of Population Sciences at TICHR. The authors have an interest in the quality of evidence that underpins current nutrition guidelines and recommendations.

## Pre-publication history

The pre-publication history for this paper can be accessed here:

http://www.biomedcentral.com/1741-7015/11/41/prepub

## Supplementary Material

Additional file 1 **Key study characteristics of identified public health weight-loss interventions**. Additional file 1 summarizes the key characteristics of identified public health interventions. This summary includes a description of the intervention; information about the study cohort, study length, and outcome measures used; and the key results. In addition, the model of energy balance apparently underpinning the intervention is identified. Click here for file
