# Supplementary material for: Tackling overweight and obesity: does the public health message match the science?
Source: BMC Med. 2013 Feb 18;11:41. doi: 10.1186/1741-7015-11-41 (PMC3626646; doi:10.1186/1741-7015-11-41)
Supplement: Additional file 1 — Key study characteristics of identified public health weight-loss interventions. Additional file 1 summarizes the key characteristics of identified public health interventions. This summary includes a description of the intervention; information about the study cohort, study length, and outcome measures used; and the key results. In addition, the model of energy balance apparently underpinning the intervention is identified. [file 1741-7015-11-41-S1.DOCX]

**Table 1.** Key study characteristics of identified public health weight loss interventions

| **First Author (reference)** | **Intervention description and key components**  (N = nutrition, PA = physical activity, B = Behavioural) | **Cohort and country** | **Study length** | **Participation** | **Key results, outcome measures, industry funding (where applicable)** | **Model of energy balance** |
| --- | --- | --- | --- | --- | --- | --- |
| **Short-term interventions (≤ 6m)** | | | | | | |
| Petrofsky [[11](#_ENREF_11)] | Compared dietary restriction and exercise program with control.  N: Energy restriction (1300-1500 cal/day) which included meal replacements.  PA: Aerobic exercise video with a 1kg mini medicine ball (60 min, 6 x wk) | Adults  (n = 123)  USA | 4wks | ≈70% | Greater weight loss in intervention group compared to control (-4.5kg vs. 0.4kg respectively). Significant changes in %BF (-2.8%), core strength and CV fitness.  Outcome measures: BMI, %BF, BP, HR, girths (waist, hips, thigh), strength | Simple  Authors make reference to energy restriction resulting in reduced basal metabolism making weight loss difficult. Suggest the inclusion of exercise in the program increases basal metabolism and the drive to regain weight is reduced. Doesn’t appear to factor into study design. |
| Shrewsbury [[18](#_ENREF_18)] | Community based weight management intervention for adolescents. Parents and adolescents attended 7 wkly group sessions which focussed on lifestyle modification.  N: Choose water as main drink, increase vegetables, keep fat intake low, choose low fat dairy foods, limit caloric content of snacks  PA: Aim to achieve 60 min/day PA, structured 20 min exercise with each wkly group session  B: Goal setting | OW - moderately OB 13-16 yo  (n = 151)  AU | 8wk results | 86% | Reduction in BMIz (-0.04), WHtR and total cholesterol. Improved perceived competence in several life domains but few changes in diet, PA, sedentary behaviour. Further assessments to be completed at 12 and 24 m.  Outcome measures: BMIz-score, WHtR, BP, metabolic profile, self-reported dietary intake, lifestyle behaviours (PA and sedentary activities), psycho-social factors | Simple |
| Kreider [[7](#_ENREF_7)] | Matched participant design which compared the effectiveness of a meal replacement based diet program which encouraged PA (MR), with a structured meal plan based diet and supervised PA (SM). Subgroup of 77 women completed a 24 wk weight maintenance phase post-intervention.  N: Energy restricted (-500 cal/day from baseline intake in MR group, 1200cal/day diet in SM group)  P: MR group encouraged to increase energy expenditure via PA, SM group completed a circuit style structured exercise program 3 x wk.  B: Incentives | OB sedentary women 18-55 yo  (n = 90)  USA | 10wk intervention and 24wk maintenance | 75% | 10wk intervention phase: SM group lost significantly more weight, fat mass and hip and waist circumference compared to the MR group. Both groups had lower resting energy expenditure (no significant difference between groups)  24wk maintenance phase: SM group maintained higher PA, weight and fat loss, greater improvements in aerobic capacity and strength.  Weight losses were small in both groups (-2.2kg and -3.1kg at 34wks for MR and SM groups respectively). No evidence of weight gain at 34 weeks  Outcome measures: height, weight, WC, bone density and body comp., REE, HR, maximal exercise capacity, 1RM, metabolic profile, PA patterns, diet record, QOL  Industry funding**:** Curves international Inc., General Mills Bell Institute of Health and Nutrition | Simple  Authors state ‘Whereas obesity  had been thought to simply be related to an imbalance  between energy intake and expenditure, more recent research  has indicated that genetic, physiological, psychological,  socioeconomic, cultural, and behavioral factors  also play a role in the etiology of obesity in various populations’ and  ‘for this reason, the prevention, treatment,  and management of obesity are complex and require  multifaceted interventions’ |
| Panagiotopoulos [[13](#_ENREF_13)] | Family centred multidisciplinary program. Included cognitive, behavioural, affective and interactional techniques for OB children and parents.  N: Education. Topics included label reading, healthy snacking, reduced SSB consumption, increased fibre and fruit and vegetable consumption, reduced portion sizes.  PA: 30 min wkly session  B: Goal setting and coping strategies | Children  (n = 119)  Canada | 10wks | 71%  63% program participation rate. | BMIz change from 2.26 to 2.2, 1.26% decrease in weight trajectory compared to pre-intervention, reduced WC and fasting insulin. Significant improvements in measures of PA and psychological measures.  Outcome measures: BMI, BMIz, WC, BP, metabolic profile, PA, psychological status. | Simple  Authors state ‘Lifestyle and behavior modification approaches have been found to be efficacious to treat childhood  obesity, at least in the short-term’ |
| Sun [[22](#_ENREF_22)] | RCT to determine the effects of an after-school exercise program with or without dietary restriction on obesity, metabolic profile and fitness in adolescents.  N: Energy restriction between 70-80% of daily standard caloric intake dependent on BMI percentile.  PA: Specific exercise training protocol (60 min, 4 x wk)  B: Education | OW adolescents  (n = 93)  China | 10wks | 100% | Significant decrease in BMI in diet and exercise group (-0.6 BMI) but not diet, exercise or control groups. BF and WC were decreased by exercise but not diet. No information about weight regains.  Outcome measures: BMI, WC, body comp., aerobic fitness, HR, metabolic profile | Simple |
| de Heer [[30](#_ENREF_30)] | Effectiveness and spill-over effects of an after-school health education and PA program. Behaviours related to obesity prevention  N: Health education  PA: Exercise goals (45-60 min PA 2 x wk) | Elementary school students  (n = 901)  USA | 12wks | 90% | -0.12, -0.11, -0.17 change in BMI for control, spill-over and intervention groups respectively. Exposure to the intervention predicted lower BMI, higher aerobic capacity and greater intention to consume a healthy diet.  Outcome measures: BMI, aerobic capacity, nutrition intentions, dietary knowledge. | Insufficient information |
| Kraschnewski [[20](#_ENREF_20)] | Evaluation of a web based weight loss intervention. Participants were matched to 3 role models who shared strategies to achieve successful weight loss. Participants self-selected which strategies to implement.  N and PA not described (varied)  B: Participants required to log into study website 1 x wk, goal setting, email reminders | OW and OB adults (n = 100)  USA | 12wks | 88% | Significantly greater weight loss in intervention group compared to controls (-1.4kg vs. 0.6kg respectively). Website participation rates were considered ‘sub-optimal’ and the authors suggested that further work was needed in order to maximise participation.  Outcome measures: Height, weight, diet, self-reported medical and weight history, weight control practices, website satisfaction. | Insufficient information. |
| Fitzgibbon [[25](#_ENREF_25)] | Obesity prevention RCT. Schools were assigned to a teacher delivered general health intervention or teacher delivered weight control intervention.  N: ‘Themed’ education sessions. Topics included portion sizes and switching to low fat products  PA: In-class exercise sessions, education to encourage movement and decrease screen time. | 3-5 yo children  (n = 618)  USA | 14wks | 81% | No significant difference between intervention and control group for change in BMI (0.11 and 0.22 respectively). Obesity prevention intervention rather than treatment.  Outcome measures: BMIz, BMI percentiles, PA (accelerometer), TV and screen time, diet. | Simple |
| Morgan, [[8](#_ENREF_8)] | Work place based RCT for male shift workers. Intervention was based on the SHED-IT program with modifications for shift workers.  N: Reduced energy intake  PA: Increased energy expenditure  B: Information sessions, program booklets, group based financial incentives and online diet and exercise records. | OW and OB men 18-65 yo  (n = 110)  AU | 14wks | 81% | Significant differences between groups for weight loss at 14wks (-4.0kg and 0.3kg weight change in intervention and control group respectively). Significant intervention effects were evident for WC, BMI, SBP, HR, PA, SSBs and PA related beliefs.  Outcome measures: weight, BMI, WC, BP, perception of the program, qualitative component | Simple  Study website provided ‘…personalized strategies to address weight loss, reduce energy intake and increase energy expenditure…’ |
| Smith West [[9](#_ENREF_9)] | 12 sessions of an adapted Diabetes Prevention Program. The lifestyle behavioural intervention was delivered in group settings by lay health educators. Controls completed a cognitive training program.  N: Goals included caloric restriction with less than 25% of calories from fat  PA: Graded goals which progressed to 150 min/wk of moderate to vigorous exercise, pedometers  B: Diet records, stimulus control, problem solving, goal setting and relapse prevention. | OB older adults  (n = 228)  USA | 16wks | 93% | Greater weight loss in intervention than in control group (-3.7kg vs. -0.3kg respectively).  Outcome measures: weight, BMI, treatment satisfaction information | Simple |
| Anderson [[4](#_ENREF_4)] | Commercial behavioural intervention program compared to usual care weight management counselling.  N: Energy restricted diet, meal replacements, focus on increasing fruit and vegetable intake  PA: PA goals (2000kcal/wk)  B: Diet record, wkly D and PA reporting, progress charts, dietician counselling | OB adults 20-65 yo  (n = 34)  USA | 6m | 69% | -16.3% vs. -1.4% change in body weight in intervention and usual care group respectively. No evidence of weight regain  Outcome measures: weight, BMI, WC, BP, metabolic profile | Simple |
| Maddison [[26](#_ENREF_26)] | RCT evaluating the effect of active video games on body weight, composition, PA and fitness. Current users of sedentary video games were assigned to control or active video games.  N: No dietary modifications  PA: Active video games | OW and OB 10-14 yo  (n = 322)  NZ | 6m | 72% | BMI change 0.09 in intervention vs. 0.34 in control. Difference was non-significant. Authors conclude a small but definitive effect on BMI and body comp. in OW and OB children.  Outcome measures: BMI, BMIz, WC, % BF, PA measures, average daily total energy consumed from snacks, time spent playing video games. | Simple  Authors state ‘Decreases in physical activity and the increased consumption of energy-dense foods are thought to contribute to these high levels of obesity’ |
| Morgan [[10](#_ENREF_10)] | Program aimed to help OW fathers lose weight and become positive role models for their children.  N: Education. Topics included energy balance, energy calculations and weight loss, food labels and recommended dietary intakes  PA: Family fitness activities, movement skills, reducing sedentary behaviours  B: Pedometers, weight loss charts, goal recording, online tracking | OW or OB men and their OW 5-12yo child  (n = 53 adults, 71 children)  AU | 6m | 66% | Significant difference between intervention fathers (-7.6kg) vs. control (0.0kg) at 6m. Intervention father’s WC, SBP, HR, and PA but not dietary intake. Significant treatment effects for PA and dietary intake for children.  Outcome measures: Weight, BMI, WC, BP, HR, PA, dietary intake | Simple |
| **Long-term interventions (> 6m)** | | | | | | |
| Hendy [[17](#_ENREF_17)] | School based weight management program. Intervention group received rewards for ‘good health behaviours’ and control group received rewards for ‘good citizenship behaviours’.  N: Eat fruits and vegetables first, choose low fat products and low sugar healthy drinks.  PA: Aim to achieve 5000 steps  B: Good health behaviours were rewarded with small rewards. | OW and normal weight children  (n = 382)  USA | 10m | 100% | -2.6 point change in BMI percentile for overweight children, -2.4 point change for average weight children (in both KCP and control). Overweight children regained lost weight after 6m post program (1.5 BMI percentile)  Outcome measures: BMI%, diet (choosing healthy drinks, exercise (steps) and consuming fruit and vegetables first) | Simple |
| Beltaifa [[5](#_ENREF_5)] | Pilot study of the use of a walk-run transition speed (WRTS) exercise training protocol in combination with dietary restriction for weight loss. 6m of dietary restriction only followed by 6m diet + WRTS exercise protocol.  N: Low energy diet 25-30% less than baseline energy intake, low fat, low GI and high fibre  PA: WRTS 3x wk increasing from 30 to 60mins per session, progressive weight training program. | OB women  (n = 37)  Tunisia | 12m | 70% | Weight loss of 11kg at 6m and total loss of 13kg at 12m. Authors concluded the addition of exercise to dietary restriction promotes a greater reduction in weight, and improvements in metabolic and cardiovascular risk factors.  Outcome measures: Weight, BMI, WC, body comp., metabolic profile, HR, WRST speed, | Simple  Discuss hormonal and hunger response to low energy diets, the compensatory changes in energy intake with increased exercise, and inclusion of a low glycaemic index diet. However, authors state ‘…the most important factors are energy restricted diets and increased physical activity.’ |
| French [[12](#_ENREF_12)] | Community based household intervention to prevent weight gain.  N: Limit high calorie snack foods, replace higher calories pre-packaged meals with lower calorie versions, limit SSB, reduce portions, ‘eat less’, limit fast food and choose healthy options when eating out.  PA: PA goals (minimum 30 min/day)  B: Daily weighing, telephone support and email contact, TV locking device in the home | 90 households  (n = 158 adults, 75 adolescents, 107 children)  USA | 12m | 97%  Low program adherence (20% attended all group sessions and home activities). | No significant intervention effect for BMIz. Intervention households reported lower levels of snacks and sweet intake, reduced spending on eating out, increased PA and self-weighing frequency.  Outcome measures: weight, height, eating behaviours, PA, TV viewing patterns | Simple |
| Golley [[28](#_ENREF_28)] | 3-arm parent led, family focussed weight management program. Children were assigned to parenting skills training plus intensive diet and activity education, parenting skills only or control.  N: Promoted dietary patterns in line with national food selection guides. Particular focus on reducing energy dense/nutrient poor products with products of lower energy density.  PA: Reduced screen time, encouraged active play and increased energy expenditure. | OW 6-9 yo  (n = 111)  AU | 12m | 95% | Intake of energy dense nutrient poor foods was lower in intervention groups at 12m compared to baseline. Reductions in screen time and active play in both intervention groups. Outcomes related to adiposity and metabolic health not included in this publication.  Outcome measures: BMIz, WCz, metabolic profile, lifestyle behaviours, QOL, body dissatisfaction, parental BMI and parental competence. | Simple  Authors provide ‘Diet, activity, behaviour modification and family support are the tools available to change energy balance and reduce adiposity in children.’ and conclude ‘…child weight management intervention that promotes food intake in line with national dietary guidelines achieves a clinically-meaningful reduction in children’s intake of energy-dense, nutrient-poor foods without compromising intake of nutrient-rich foods. |
| Morgan [[27](#_ENREF_27)] | Internet based weight loss program for men (SHED-IT). Program used Calorie King health website to track diet and exercise. Participants were provided with education and feedback on how to reduce energy intake, and increase expenditure.  N: Reduced energy intake  PA: Increased energy expenditure.  B: Information sessions, program booklets, group based financial incentives and online diet and exercise records. | OW and OB men  (n = 65)  AU | 12m | 71% | Significant and sustained weight loss at 12 m for intervention (-5.3kg) and controls (-3.1kg). No significant differences between groups. Significant group by time interaction for weight, WC, BMI and SBP.  Outcome measures: weight, BMI, WC, BP, process evaluation | Simple  Authors state ‘…respondents commented on the value of the information session in terms of the motivational, clear, simple messages presented, and in particular the simplicity of the energy in/ energy out formula resonated with participants…’ |
| Collins [[6](#_ENREF_6)] | RCT which compared the combination of a child focused PA program plus parent centred dietary modification program with either of the programs alone.  N: Reduced total energy and fat intake, increased fruit and vegetable intake, healthy beverage and snack choices  PA: Wkly structured exercise aimed at improving fundamental movement skills  B: Goal setting with regular follow-up | OW 5-10 yo children  (n = 165)  AU | 24m | 80% | Change in BMIz at 24m: Activity and diet (-0.24), activity only (-0.19), diet only (-0.35). All groups reduced BMIz. Parent-centred diet program resulted in the greatest weight loss. Activity and diet, and diet only groups showed a greater reduction than activity alone. No significant differences between groups for WC, WtHR, metabolic outcomes. Evidence of weight regain after 12m in all groups.  Outcome measures: BMIz, height, weight, WC, BP, metabolic profile, energy intake, PA  Industry funding: Sanitarium Health Food Company | Simple |
| Llargues [[24](#_ENREF_24)] | Cluster randomised prospective obesity prevention program. Intervention evaluated the promotion of healthy eating habits and PA using the education methodology; ‘Investigation, Vision, Action and Change’.  N: Education regarding healthy diet, healthy recipes provided to families  P: Education, encouragement of at school and weekend PA.  B: Nutrition and PA education provided to families | 5-6 yo children  (n = 598)  Spain | 24m | 72% | BMI change 0.8 vs. 1.8 intervention and control respectively.  BMI of control group was 0.89 higher than that of the intervention group. Prevalence of OW children was decreased by 62%. Increased fruit intake and after-school PA in intervention group.  Outcome measures: BMI, height, PA, dietary factors, parental BMI | Simple  Authors state the ‘increase in obesity can be attributed to… an imbalance between the intake and the expenditure of energy’ |
| Rock [[23](#_ENREF_23)] | RCT which compared an incentivised structured weight loss program (telephone or centre based) with usual care.  N: Intervention focussed on low fat, reduced energy density, calorie restricted diet (1200-2000 kcal/day). 500-1000 kcal/day reduction in dietary intake in usual care group.  PA: Increased PA to achieve 30 mins on 5 or more days per wk, PA goals.  B: Counselling, educational materials provided | OW or OB women 25-40 yo  (n = 442)  USA | 24m | 92% | Structured weight loss program resulted in greater weight loss than the usual care group at 12m (-10kg vs. -8.5kg) and 24m (-7.4kg vs. -6.2kg). Usual care group had an average weight change of -2.4kg and -2kg at 12 and 24m respectively. At 24m centre based, telephone based and usual care had significantly reduced weight from baselines.  Evidence of weight regain in both centre and telephone based groups from 12m.  Outcome measures: height, weight, WC, QOL, metabolic profile,  Industry funding: Jenny Craig Inc. | Simple |
| Shofan [[31](#_ENREF_31)] | School based intervention which focused on increased physical education and activity and nutritional advice for children and their families with the aim of preventing obesity. Control group received standard physical education lessons only.  N: 8 nutritional education lessons over 2yrs, parent meetings to encourage healthy dietary habits  PA: Double PA education hours compared to controls | 9 yo children  (n = 65)  Israel | 24m | 90% | Study group reduced their average BMI percentile by approximately 6% at the end of the intervention. Boys in the control group gained significantly more weight than those in the intervention group. There was no significant change in the weight or BMI in girls. No significant change in BMI of the control group (0.48). No changes in the PA, fruit and vegetable intake, fast food consumption and sedentary behaviour in intervention group.  Outcome measures: weight, height, BMI%, dietary factors, PA | Simple  Despite no difference in the diet or PA habits of the children post-intervention the authors conclude that ‘…an all around intervention including both physical fitness and nutritional classes…would give the best results.’ |
| Stark [[15](#_ENREF_15)] | Pilot clinic and home based behavioural intervention was compared to enhanced standard care involving paediatrician counselling. Intervention focused on healthy eating activities for parents and children.  N: Energy restricted 1000-1200 calorie intake, reduced SSB, limited portion sizes and eating out, promoted fruit and vegetable consumption.  PA: PA goals, pedometers, reduced sedentary behaviour and promoted active play.  B: Dietary education, PA and parenting skills | 2-5 yo and an OW parent  (n = 36)  USA | 12m | 89% | Significantly greater reductions in child BMz, BMI percentile, and weight gain after 6m program which were maintained at 12m follow up. 12m change in BMIz -0.37 and 0.4 for intervention and paediatrician counselling respectively. Intervention parents had greater weight loss than those in the control. Evidence of weight regain at 12m in 3 of 7 children in the intervention group  Outcome measures: weight, height, BMIz, BMI percentile, dietary recall, home food environment, PA | Simple |
| Tucker [[14](#_ENREF_14)] | Non-randomised quasi experimental design with control and intervention group. ‘Let’s go 5-2-1-0 Program’ involved coaching by nursing students on health parameters for young children.  N: Focused on eating fruit and vegetables, restricting SSBs limitation of sport and fruit drinks  PA: PA goals (min 60min of moderate PA every day, 10 mins of vigorous activity 3 x wk), pedometers  B: Motivational interviewing | 4-5^th^ grade children from 2 schools  (n = 99)  USA | 12m | 99% | Significant decrease in BMI percentile in one school (-6.2) at 12m. No significant difference in second school  Outcome measures: BMI, BMI%, TV viewing, healthy habits (diet, PA behaviours) | Simple |
| Tan-Ting [[21](#_ENREF_21)] | Hospital based combined diet, exercise and behavioural therapy.  N: 1200-1500 calorie energy restricted diet  PA: Structured exercise (3 x 1 hour sessions per wk), exercise goals (30mins/day increased by 10 mins each time goal was achieved).  B: Psychiatrist support | OB children  (n = 44)  The Philippines | 15m | 100%  Low adherence to program 29.5% of participants completed less than 12 sessions, 59% completed 12-24 sessions and 13.6% completed all 24 sessions | Significant reductions in weight (- 4.2kg), BMI (-1.5) BMIz (-0.15) %BF (-14%)  Outcome measures: BMI, BMIz, body comp., BP, WC, hip circumference | Simple  Authors note factors contributing to obesity are often over simplified and state ‘Obesity appears to be a multifactorial condition; hence, a multidisciplinary approach is often advocated for its management’. |
| Mitchell [[19](#_ENREF_19)] | Evaluation of the weight loss success of participants involved in a non-profit weight loss program called Take Off Pounds Sensibly (TOPS)  N and PA: Program participants received information regarding healthy eating based on national recommendations, exercise and behaviour modification. An individual’s regime was self-initiated and therefore no additional information was provided.  B: Group meetings and/or online support | Adults  (n = 42 481)  USA | 24-36m | NA | Participants who stayed in the program lost 5.9–6.8% of initial weight in the first yr and maintained that weight loss over the following 24m. Low membership renewal (37% at 1 year) and therefore results are unlikely to reflect true weight loss success.  Outcome measures: weight | Simple  No individual dietary or PA data provided but recommendations provided to participants appeared to be based on a simple model. |
| Millar [[16](#_ENREF_16)] | Intervention focused on building the capacity of families, schools and communities to promote healthy eating and PA.  N: Reduced SSB consumption, promotion of healthy breakfast, increased fruit and vegetable consumption, traffic light labelling in schools.  PA: Promoted participation in active transport, active recreation and organised sports. | 12-18 yo and their families  (n = 2054 children)  AU | 36m | 67% | Change in BMIz and body weight were significant but small (-0.07 and -0.74kg respectively). No significant reduction in the prevalence of OW and obesity. Little change in positive dietary and PA measures.  Outcome measures: height, weight, BMI, BMIz, body comp., QOL | Simple |

Abbreviations : AU – Australia, B – behavioural, %BF – percentage body fat, BMI – body mass index, BMIz – Body mass index z-score, body comp. – body composition, BP – blood pressure, Cal – calorie, DBP – diastolic blood pressure, GI – glycaemic index, m - month, N – nutrition, NZ – New Zealand, OB – obese, OW – overweight, PA – physical activity, RCT – randomised control trial, HR – heart rate, QOL – quality of life, 1RM – one repetition max, SBP – systolic blood pressure, SSB – sugar sweetened beverage, USA – United States of America, WC – waist circumference WCz – waist circumference z score, wk - week, WtHR – waist to hip ratio, yr – year.
